# Poverty and Mortality Risk in Patients With Colorectal Cancer

**DOI:** 10.1001/jamanetworkopen.2026.6303

**Published:** 2026-04-10

**Authors:** Mario Schootman, Chenghui Li, Cheng Peng, Peter DelNero, Emily Hallgren, Zhengmin Qian, Jonathan Laryea

**Affiliations:** 1 Winthrop P. Rockefeller Cancer Institute, University of Arkansas for Medical Sciences, Little Rock,; 2 Department of Internal Medicine, College of Medicine, University of Arkansas for Medical Sciences, Little Rock; 3 College of Nursing, Little Rock, Arkansas; 4 Division of Pharmaceutical Evaluation and Policy, College of Pharmacy, University of Arkansas for Medical Sciences, Little Rock; 5 Department of Health Policy and Management, College of Public Health, University of Arkansas for Medical Sciences, Little Rock; 6 Department of Medicine, Larner College of Medicine, University of Vermont, Burlington; 7 Department of Epidemiology and Biostatistics, College for Public Health and Social Justice, Saint Louis University, St Louis, Missouri; 8 Department of Surgery, College of Medicine, University of Arkansas for Medical Sciences, Little Rock

## Abstract

**Importance:**

Colorectal cancer (CRC) is the third most common cancer in the US, accounting for 9% of all cancer deaths. People in persistent poverty areas—where at least 20% have lived in poverty for at least 30 years—face higher colorectal cancer mortality than those in other areas.

**Objective:**

To investigate which risk factors mediated the association between living in persistent poverty areas and mortality, specifically disease severity and aggressiveness, type of CRC treatment, quality of treatment, access to medical care, and complications.

**Design, Setting, and Participants:**

This retrospective cohort study used linked data from the Central Cancer Registry, Death Certificates, and the All-Payer Claims Database (APCD) from January 2013 to June 2023. The population included patients from urban census tracts in Arkansas who were newly diagnosed with colorectal cancer. Analyses were conducted between May and September 2025.

**Exposure:**

Residence in a persistent poverty census tract.

**Main Outcome and Measures:**

The main outcome was overall survival. Potential confounders (including demographics and comorbidities) and potential mediators (including disease severity and aggressiveness, type and quality of treatment, health care access, and complications) were also examined.

**Results:**

Among 5028 patients newly diagnosed with CRC in 382 urban census tracts, 2587 (51.5%) were male; 705 (14.0%) were Black, 4142 (82.4%) were White, and 181 (3.6%) were of other race or ethnicity; and 2371 (47.2%) were married, with a mean (SD) age of 64.6 (13.7) years. Among 617 patients living in persistent poverty tracts, 329 (53.3%) died, vs 1927 of 4411 (43.7%) in other tracts (hazard ratio, 1.17; 95% CI, 1.03-1.33). There was significant evidence of mediation by stage at diagnosis (33.7% mediation; 95% CI, 7.4%-89.5%; *P* = .01), not having surgery (29.3% mediation; 95% CI, 5.5%-87.2%; *P* = .02), and type of health insurance (13.8% mediation); 95% CI, 2.2%-55.3%; *P* = .03).

**Conclusion and Relevance:**

In this retrospective cohort study of patients with CRC in urban persistent poverty areas, more advanced stage, not receiving surgery, and type of health insurance were key mediators of their increased risk of mortality. Improved awareness of these mediators may help inform targeted interventions to reduce the risk of mortality in this population.

## Key Points

**Question:**

Which risk factors mediate the association between living in persistent poverty areas and increased mortality?

**Findings:**

In this cohort study that included 5028 patients newly diagnosed with colorectal cancer in 382 urban census tracts, there was significant evidence of mediation by stage at diagnosis (33.7% mediation), not having surgery (29.3% mediation), and type of health insurance (13.8% mediation).

**Meaning:**

These findings suggest that investments are needed to improve screening and subsequent follow-up and to facilitate the receipt of surgery, financial assistance, and other patient support programs, which may reduce the increased risk of mortality among patients with colorectal cancer in urban persistent poverty areas.

## Teaser

This cohort study examines which risk factors mediate the association between living in persistent poverty areas and mortality and treatment outcomes among people with colorectal cancer in the US.

## Introduction

Colorectal cancer (CRC) is the third most common cancer in the US, accounting for 9% of all cancer deaths.^1^ People in persistent poverty areas—where at least 20% have lived in poverty for at least 30 years—face higher CRC mortality rates than those in other areas.^2,3^ Understanding the impact of persistent poverty on CRC mortality is crucial because persistent poverty is strongly linked to various socioeconomic conditions, such as limited education, unstable housing, inadequate nutrition, and restricted access to high-quality health care.^4^ These interconnected factors often create a vicious cycle in which cancer patients residing in persistently impoverished areas face poorer health outcomes and higher medical expenses, further entrenching them in poverty.^5^

There is a need to understand why those living in persistent poverty areas have a worse prognosis and to identify opportunities to reduce their risk of dying following CRC diagnosis.^6,7^ This is especially important for states significantly impacted by both CRC and persistent poverty, such as Arkansas. Arkansas has the fifth-highest age-adjusted CRC mortality rate nationally,^2^ and the prevalence of residents living in persistent poverty areas exceeds the national average by about 50%.^8^ Those diagnosed with CRC and who live in persistent poverty areas are at an increased risk of dying compared with those living elsewhere in Arkansas,^9^ especially those in urban areas. Median survival was 4.5 years shorter for those living in urban persistent poverty census tracts compared with urban nonpersistent poverty tracts. This difference is worse than in other parts of the United States when comparing persistent poverty and nonpersistent poverty areas.^2,10^

Several plausible mechanisms, rooted in known mortality risk factors, may explain the elevated risk of death among those diagnosed with CRC living in areas of persistent urban poverty.^8,11,12,13,14^ This study investigated the extent to which individual-level risk factors mediated the association between living in persistent poverty areas and mortality,^15^ specifically disease severity and aggressiveness, type of CRC treatment, quality of treatment, access to medical care, and complications. Identifying specific mediators will help target interventions among patients with CRC living in urban areas with persistent poverty.

## Methods

### Data Source

In this retrospective cohort study, we used the Arkansas All-Payer Claims Database (APCD) and its linked databases from the Arkansas Department of Health (Arkansas Central Cancer Registry [ACCR] and Death Certificate Database), as well as census tract–level census data. The APCD database included commercial and Medicaid claims from January 2013 to June 2023 and Medicare claims from 2013 to 2020. The linked ACCR 2013-2019 and Death Certificate databases were from 2013 to 2022. Eligibility files were obtained from private and public payers in Arkansas. A unique alias (patient ID) based on a person’s last name, birth date, and sex was used to link patients across databases. State APCDs capture the longitudinal health care utilization of all but a small minority of patients (eg, those covered by the Veterans Administration) across various settings and age groups, providing a more comprehensive picture of care, even when patients switch insurance plans or are covered concurrently by multiple payers.^16^ Coverage of the APCD was comparable between persistent and nonpersistent areas, resulting in minimal bias in this study.^16,17^ This study followed the Strengthening the Reporting of Observational Studies in Epidemiology (STROBE) reporting guideline and was approved by the University of Arkansas for Medical Sciences institutional review board, which approved it as exempt because of the use of existing, deidentified data.

### Patient Selection

Patients with a first primary CRC diagnosis between 2013 and 2019 were identified from the Arkansas Cancer Registry using *International Classification of Diseases for Oncology, 3rd Edition* codes (C18-C20, excluding C18.1 [appendix cancer] and histology codes 9050-9055, 9140, and 9590-9993). We included patients in urban areas only because those diagnosed with CRC who lived in urban persistent poverty areas were at increased risk of dying compared with those living in other urban areas.^9^ The patient’s census tract at the time of diagnosis was defined using the US Department of Agriculture (USDA) Rural-Urban Commuting Area (RUCA) codes, based on the 2010 decennial census and 2006-2010 American Community Survey.^18^ Tracts were classified as urban if located in metropolitan areas (RUCA codes 1-3) or if the commuting flow to urban areas was at least 30% (RUCA codes 4.1, 5.1, 7.1, 8.1, and 10.1), as specified in the ACCR data. Patients from all other tracts were excluded.

Patients were excluded if they (1) had a prior history of cancer; (2) had discrepancies between ACCR and death records; (3) were missing information for census tract, sex, or date of diagnosis; (4) were identified from death certificate or autopsy only; (5) died on the same date as the date of diagnosis, meaning survival time could not be calculated; (6) had in-situ disease; (7) resided in tracts where persistent poverty status was not available; or (8) had a patient ID that matched multiple unique beneficiaries.

In the main analysis, we included only mediators from the ACCR. The secondary analysis included a subset of patients with continuous insurance coverage in APCD 6 months before and 12 months after diagnosis or until the date of death if it occurred within 12 months (continuous coverage cohort). The mediators identified from the AACR were also examined in the secondary analysis.

### Persistent Poverty Status

We used census tracts designated as persistent poverty to allow a more fine-grained analysis of long-term concentrated poverty. Persistent poverty tracts were identified by the 2019 estimates of the USDA as having poverty rates of at least 20.0% for 4 consecutive measurement periods spanning 30 years (decennial census 1-year estimates for 1990 and 2000 and American Community Survey 5-year estimates for 2007-2011 and 2015-2019). Persistent poverty status was not available for 29 of the 686 census tracts (4.2%) in Arkansas if poverty rate estimates were missing for 1 or more measurement periods or if the estimated margin of error included 20.0% and the reliability index was low.

### Patient Outcome

The primary outcome was overall survival. All-cause mortality was operationalized as death following CRC diagnosis using vital status in the ACCR supplemented with death certificates. We also describe CRC-specific deaths as well as deaths from other causes.

### Potential Mediators and Confounders

**Figure 1.**
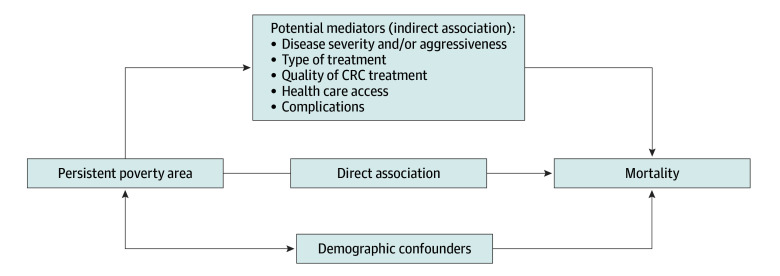
Conceptual Model of Potential Mediators and Confounders for the Association Between Living in a Persistent Poverty Area and Risk of Mortality Following Colorectal Cancer A double-headed arrow refers to an association; a single-headed arrow refers to variables associated with the risk of mortality.

Potential mediators and confounders were based on studies of prognostic factors of mortality among persons living in persistent poverty areas or those diagnosed with CRC (Figure 1). The confounders are associated with both persistent poverty areas and mortality; however, they are not part of the causal pathway and are not amenable to intervention (Figure 1). Potential confounders included patient demographic characteristics (sex, age group, and race and ethnicity) as well as comorbidities at the time of diagnosis. Central cancer registries collect race and ethnicity data from patients’ medical records. Race and ethnicity were included as confounders as imperfect proxies for systemic factors, structural inequities, and other unmeasured confounders. The National Cancer Institute comorbidity index was calculated using diagnosis codes from 6 months preceding CRC diagnosis using the APCD.^5^

Mediators are variables hypothesized to be in the causal pathway between persistent poverty areas and mortality and must be associated with both. The exposure (residing in a persistent poverty area) is presumed to cause the mediator, and the mediator is presumed to cause the outcome, not vice versa.^19^ Potential mediators were disease severity and aggressiveness, type of treatment, quality of CRC treatment, health care access, and complications. Disease severity and aggressiveness included stage at diagnosis, lymphovascular invasion, tumor location, and tumor grade, and were obtained from the ACCR. Type of treatment included type of surgery, radiation therapy, and chemotherapy. The type of surgery was extracted from the ACCR. We included claims up to 6 months after diagnosis to identify initial treatment. To assess receipt of radiation therapy, we used the APCD files.^20,21^ A single claim was considered indicative of receiving radiation therapy. The radiation therapy had to be within 120 days of the date of the first surgical claim.^22^ We used chemotherapy drugs for CRC.^23,24^ A single claim was considered indicative of chemotherapy. This method also captures neoadjuvant chemotherapy. Chemotherapy medications were identified in claims data using Health Care Common Procedure Coding System codes. Quality of CRC treatment included the number of lymph nodes examined and 30-day readmission, obtained from the ACCR. Readmission was defined as any readmission for any reason to the same hospital within 30 days of the date of discharge. For health care access, we identified through the ACCR the primary type of health insurance obtained at the time of diagnosis. Complications included anastomotic leak (AL) and venous thromboembolism (VTE). There are no specific medical codes to identify ALs. The occurrence of AL was approximated by the presence of at least 1 of the *International Classification of Diseases, Ninth Revision* or *International Statistical Classification of Diseases and Related Health Problems, Tenth Revision* diagnosis codes of infection, peritonitis, septicemia, or abscess within 30 days after the surgery date, recognizing that not all were the result of an AL.^25^ These cases also needed to show the presence of at least 1 of the following procedures that are indicative of the AL intervention: (1) laparotomy, (2) incision of the abdominal wall, and (3) requirement of drainage during the index stay. Data about VTE and AL were obtained from the APCD (eAppendixes 1 and 2 in Supplement 1).^26,27^

### Statistical Analysis

Analyses were conducted between May and September 2025. First, we provided descriptive statistics for patients in the persistent and nonpersistent poverty areas. Second, we tested the associations between persistent poverty status and each potential mediator and confounder using χ^2^ tests for categorical variables. Third, we identified the confounders of the association between persistent poverty status and mortality or between a mediator and mortality for inclusion in the mediator models using accelerated failure-time regression analysis.^28^ Confounders were included if they altered the hazard ratio (HR) for the association between persistent poverty and risk of death by at least 10%. Fourth, we determined the interaction between persistent poverty status and the potential mediators.^29^ Fifth, we identified potential mediators associated with persistent poverty status and mortality, adjusting for confounders, and reported HR and 95% CIs. Mediators are variables hypothesized to be in the pathway between persistent poverty areas and mortality and must be associated with both.^19^ We used accelerated failure-time models with a Weibull distribution, which allow for estimation of covariate associations on survival time. Sixth, we employed causal mediation analysis using the mediation macro to determine the extent to which each variable mediated the association between persistent poverty status and mortality in separate single mediator models.^30^ This macro is designed to implement mediation analysis in the presence of exposure-mediator interaction, accounting for different types of outcomes (normal, dichotomous, Poisson, negative binomial, failure time) and mediators of interest. A causal interpretation of mediation analysis estimates requires that confounders suffice to control for confounding for the persistent associations between poverty status and mortality, mediator and mortality, and persistent poverty status and mediator.^31^ The proportion mediated for each mediator was calculated as the indirect effect divided by the total effect. In causal mediation analysis, the indirect effect represents the portion of the total effect of an exposure (ie, persistent poverty) on an outcome (ie, mortality) that operates through a mediator (Figure 1). We also reported the *P* value for the indirect effect.^31^ We estimated 95% CIs for the proportion mediated by using Monte Carlo simulation, drawing from normal distributions defined by the log-transformed natural indirect and total effects and their standard errors. A *P* value cutoff of <.05 indicated statistical significance. All data management and statistical analyses were performed using SAS, version 9.4 (SAS Institute, Inc., Cary, North Carolina).

## Results

**Figure 2.**
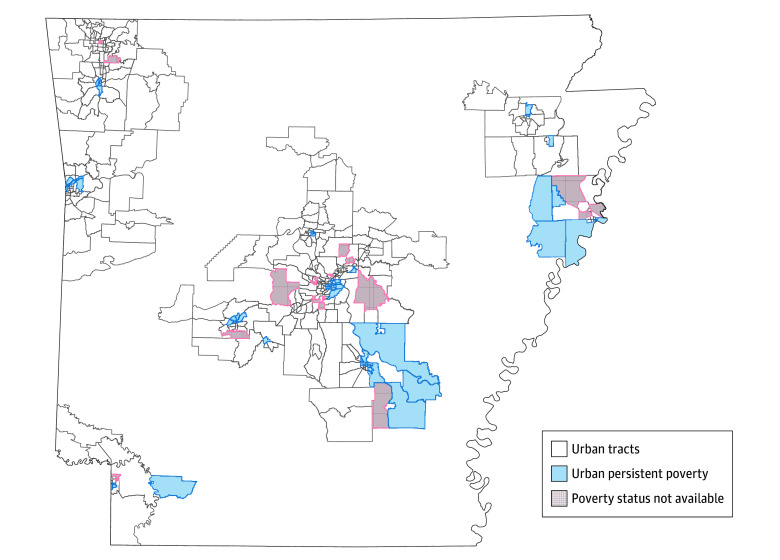
Urban Census Tracts in Persistent Poverty and Nonpersistent Poverty in Arkansas During the study period, 5028 patients were newly diagnosed with colorectal cancer in 382 urban census tracts in Arkansas.

We identified 10 692 patients diagnosed with CRC. Using the selection criteria, we excluded 2699 patients (eFigure in Supplement 1), leaving 2965 rural and 5028 urban patients. During the study period, 5028 patients were newly diagnosed with CRC in 382 urban census tracts in Arkansas (Figure 2). Of these patients, 2441 (48.5%) were female and 2587 (51.5%) were male; 705 (14.0%) were Black, 4142 (82.4%) were White, and 181 (3.6%) were of other race or ethnicity (including American Indian or Alaska Native, Asian, Native Hawaiian or Other Pacific Islander, and unknown/not stated); and 2371 (47.2%) were married, with a mean (SD) age of 64.6 (13.7) years. Table 1 shows that 617 patients with CRC (12.3%) lived in persistent poverty urban tracts at the time of diagnosis. Patients who lived in these tracts were more likely to be Black, be diagnosed with distant CRC, and have Medicaid insurance. Patients who lived in persistent poverty urban tracts were less likely to be married, less likely to receive surgery, and more likely have fewer lymph nodes examined (<12 vs ≥12) than those living in nonpersistent poverty urban tracts. None of the variables (sex, age, race and ethnicity, CRC diagnosed before 50 years of age, marital status, and comorbidity) acted as confounders among all 5028 patients, as the HR for persistent poverty did not change by more than 10% when each variable was included in the accelerated failure-time models. Many of the potential mediators increased the risk of death when controlling for persistent poverty (Table 1). Characteristics that could not be mediators because they were not associated with persistent poverty and survival included disease severity and aggressiveness (lymphovascular invasion, tumor location), treatment type (radiation, chemotherapy), quality of treatment (readmission), and complications (VTE, anastomotic leak). Compared with patients without continuous coverage, the group with continuous coverage had a lower percentage of males, patients diagnosed at younger than 50 years, Black individuals, and married patients (eTables 1 and 2 in Supplement 1). Patients with continuous coverage also had a lower percentage of local staged cancers, rectal cancer, radiation therapy, and 12 or more lymph nodes examined. However, they had a higher percentage of chemotherapy, Medicare patients, and anastomotic leakage.

**Table 1.** Study Population Characteristics in Associations of Each Potential Confounder and Mediator With Persistent Poverty Status and Risk of Death Among Urban Patients With Colorectal Cancer, for the Main Cohort and the Continuous Coverage Cohort

Characteristic	Poverty, No. (%)			*P* value^a^	HR of each potential confounder and mediator with survival, adjusted for persistent poverty (95% CI)
	Persistent	Nonpersistent	Total		
**Main cohort**					
No.	617	4411	5028		NA
Potential confounders					
Sex					
Male	313 (50.7)	2274 (51.6)	2587 (51.5)	.70	1.10 (1.01-1.19)
Female	304 (49.3)	2137 (48.4)	2441 (48.5)		1 [Reference]
Age, mean (SD), y	64.8 (12.6)	64.6 (13.8)	64.6 (13.7)	.67	1.03 (1.02-1.03)
CRC diagnosis at age <50 y	67 (10.9)	595 (13.5)	662 (13.2)	.07	0.70 (0.62-0.79)
Race and ethnicity					
Black	263 (42.6)	442 (10.0)	705 (14.0)	<.001	0.87 (0.70-0.99)
White	317 (51.4)	3825 (86.7)	4142 (82.4)		1 [Reference]
Other^b^	37 (6.0)	144 (3.3)	181 (3.6)		0.63 (0.37-0.81)
Marital status (married)	209 (33.9)	2162 (49.0)	2371 (47.2)	<.001	0.85 (0.79-0.92)
Potential mediators					
Disease severity and aggressiveness					
Stage					
Local	171 (27.7)	1149 (26.1)	1320 (26.3)	.005	1 [Reference]
Regional	186 (30.2)	1505 (34.1)	1691 (33.6)		1.55 (1.34-1.81)
Distant	149 (24.2)	835 (18.9)	984 (19.6)		5.98 (4.91-7.38)
Unknown	111 (18.0)	922 (20.9)	1033 (20.5)		1.77 (1.51-2.10)
Lymphovascular invasion					
Yes	130 (21.1)	1063 (24.1)	1193 (23.7)	.07	2.02 (1.77-2.32)
No	262 (42.5)	1925 (43.6)	2187 (43.5)		1 [Reference]
Unknown	225 (36.5)	36.5 (32.3)	1648 (32.8)		2.11 (1.86-2.42)
Tumor grade					
Well-differentiated	50 (8.1)	234 (5.3)	284 (5.7)	.01	1 [Reference]
Moderately differentiated	270 (43.8)	1995 (45.2)	2265 (45.1)		1.49 (1.19-1.88)
Poorly differentiated	45 (7.3)	467 (10.6)	512 (10.2)		2.42 (1.87-3.21)
Undifferentiated	3 (0.5)	23 (0.5)	26 (0.5)		1.70 (0.93-3.25)
Unknown	249 (40.4)	1692 (38.4)	1941 (38.6)		2.00 (1.58-2.57)
Tumor location					
Colon	438 (71.0)	3104 (70.4)	3542 (70.5)	.75	1 [Reference]
Rectum	179 (29.0)	1307 (29.3)	1486 (29.6)		0.89 (0.82-0.98)
Treatment type					
Type of surgery					
No surgery	124 (20.1)	705 (16.0)	829 (16.5)	.04	3.29 (2.86-3.82)
Local excision	43 (7.0)	244 (5.5)	287 (5.7)		0.45 (0.36-0.57)
Partial, total colectomy, proctocolectomy	425 (68.9)	3274 (74.2)	3699 (73.6)		1 [Reference]
Palliative	3 (0.5)	31 (0.7)	34 (0.7)		1.12 (0.69-1.88)
Unknown	22 (3.6)	157 (3.6)	179 (3.6)		1.11 (0.88-1.43)
Quality of treatment					
Lymph nodes examined^c^					
≥12	307 (62.3)	2553 (68.9)	2860 (68.1)	.001	1.06 (0.95-1.20)
<12	159 (32.3)	977 (26.4)	1136 (27.1)		1 [Reference]
Unknown	27 (5.5)	176 (4.8)	203 (4.8)		0.88 (0.69-1.15)
Healthcare access					
Health insurance					
Uninsured	23 (3.7)	124 (2.8)	147 (2.9)	<.001	1.56 (1.21-2.05)
Medicaid only	65 (10.5)	264 (6.0)	329 (6.5)		1.46 (1.21-1.79)
Medicare	263 (42.6)	1944 (44.1)	2207 (43.9)		1.73 (1.54-1.96)
Private	187 (30.3)	1669 (37.8)	1856 (36.9)		1 [Reference]
Medicare and Medicaid	44 (7.1)	191 (4.3)	235 (4.7)		2.07 (1.68-2.59)
Unknown	35 (5.7)	218 (4.9)	253 (5.0)		1.10 (0.89-1.38)
**Continuous coverage cohort**					
No.	270	1970	2240		NA
Potential confounders					
Comorbidity					
None	68 (25.2)	650 (33.0)	718 (32.1)	.01	1 [Reference]
1	66 (24.4)	479 (24.3)	545 (24.3)		1.79 (1.46-2.23)
≥2	118 (43.7)	734 (37.3)	852 (38.0)		2.66 (2.16-3.33)
Unknown	18 (6.7)	107 (5.4)	125 (5.6)		1.28 (0.94-1.81)
Potential mediators					
Treatment type					
Radiation					
Yes	25 (9.3)	220 (11.2)	245 (10.9)	.64	0.78 (0.65-0.96)
No	220 (81.5)	1621 (82.3)	1841 (82.2)		1 [Reference]
Unknown	25 (9.3)	129 (6.6)	154 (6.9)		0.77 (0.61-0.99)
Chemotherapy					
Yes	105 (38.9)	787 (40.0)	892 (39.8)	.38	1.10 (0.98-1.26)
No	165 (67.9)	1183 (60.1)	60.2		1 [Reference]
Quality of treatment					
Readmission within 30 d					
Not readmitted	77.8	83.7	83.0	.16	1 [Reference]
Unplanned	3.7	3.1	3.1		1.40 (1.00-2.06)
Planned	3.0	1.0	3.1		1.10 (0.64-2.03)
Unknown	14.4	10.3	12.7		1.04 (0.85-1.31)
Complications					
Venous thromboembolism	5.6	3.6	3.8	.04	2.29 (1.62-3.38)
Anastomotic leakage	11.5	11.3	11.2	.02	1.65 (1.48-2.40)

Abbreviations: HR, hazard ratio; NA, not applicable.

^a^ *P* values are for the comparison of potential confounders or mediators between patients with colorectal cancer living in persistent poverty tracts and those who lived in nonpersistent tracts.

^b^ Includes American Indian or Alaska Native, Asian, Native Hawaiian or Other Pacific Islander, and unknown or not stated.

^c^ Among those who had surgery (n = 4199).

Overall, 2772 patients with CRC were alive at the end of the study period (55.1%), 1378 died from CRC (27.4%), and 878 (17.5%) died from other causes (Table 2). Among the 617 in persistent poverty tracts, 329 (53.3%) died, vs 1927 of 4411 (43.7%) in other tracts. The percentages of both CRC and non-CRC deaths were higher in persistent poverty tracts compared with other tracts. The mean (SD) length of follow-up was 1636 (1054) days (range, 1-3651 days). Overall, the hazard of death was higher for patients with CRC who lived in persistent poverty tracts than for patients who lived in other tracts in unadjusted analysis (HR, 1.17; 95% CI, 1.03-1.33).

**Table 2.** Vital Status by Persistent Poverty Location of Patients With Colorectal Cancer

Status	No. (%)			
	Persistent poverty	Nonpersistent poverty	Total
Died	329 (53.3)	1927 (43.7)	2256 (44.9)
Colorectal cancer	195 (31.6)	1183 (26.8)	1378 (27.4)
Other causes	134 (21.7)	744 (16.9)	878 (17.5)
Alive	288 (46.7)	2484 (64.6)	2772 (55.1)
Total	617 (100)	4411 (100)	5028 (100)

Table 3 shows that 3 ACCR variables significantly mediated the association between persistent poverty and risk of death. There was significant evidence of mediation by stage at diagnosis (33.7% mediation; 95% CI, 7.4%-89.5%; *P* = .01) when dichotomized as local or regional vs distant stage. Not having surgery mediated the association for 29.3% (95% CI, 5.5%-87.2%; *P* = .02). There was no difference in having surgery between those living in persistent poverty areas vs those living elsewhere among those diagnosed with local/regional disease and among those with metastatic disease. This was also true among those diagnosed with colon cancer and those diagnosed with rectal cancer. A higher percentage among those who lived in persistent poverty areas with private health insurance did not have surgery (39 of 178 [21.7%]), vs those who lived elsewhere with this same type of insurance (371 or 1622 [14.1%]) (*P* = .001). There was also evidence of mediation for health insurance (13.8% mediation; 95% CI, 2.2%-55.3%; *P* = .03) when dichotomized as private vs other types of insurance. There were no statistically significant interactions between persistent poverty status and any of the potential confounders (sex, age, race and ethnicity, marital status, and comorbidity), suggesting that the mediation effects did not vary by sex, age, race and ethnicity, marital status, and comorbidity. None of the APCD variables were mediators in the continuous coverage cohort (eTable 3 in Supplement 1). In a sensitivity analysis with listwise deletion of missing mediator data from Table 3, the results were very similar.

**Table 3.** Proportion of the Association Between Persistent Poverty and Risk of Death Mediated by Stage at Diagnosis, Having Surgery, and Health Insurance

Mediator	% Mediated (95% CI)	*P* value for the indirect effect	No.
Stage at diagnosis	33.7 (7.4-89.5)	.01	3995
Surgery performed	29.3 (5.5-87.2)	.02	4849
Type of health insurance	13.8 (2.2-55.3)	.03	5028

## Discussion

The purpose of this cohort study was to identify mediators that explain the increased risk of dying among CRC patients in urban persistent poverty census tracts. There was significant evidence of mediation by stage at diagnosis, type of surgery, and type of health insurance. Identification of significant mediators can help inform targeted interventions aimed at reducing this elevated risk.

Unlike previous conflicting results,^32,33^ stage at diagnosis varied across persistent poverty areas and was a significant mediator in this study. There could be several reasons for a more advanced stage at diagnosis among patients with CRC in persistent poverty census tracts, including a delay in diagnosis (time between symptoms and consultation with a physician or between physician consultation and diagnostic resolution), lack of screening,^34,35^ lack of appropriate and timely follow-up after a positive screening, false negative screening, and development of cancer after a negative screen. Given the available data in the AACR, it is not possible to disentangle these variables and determine the reason for the more advanced stage among those living in persistent poverty areas. The use of self-reported or electronic medical record data may provide additional insight for future intervention development and implementation.

Our results also showed that whether a patient had surgery played a role. A higher percentage of patients with CRC living in persistent poverty areas did not have surgery vs those living elsewhere, which was previously observed.^32^ This was not the result of being diagnosed with a more advanced stage or with colon vs rectal cancer. However, a higher percentage of those living in persistent poverty areas with private health insurance did not undergo surgery (21.7%) compared with those living elsewhere with the same type of insurance (14.1%). This may be the result of higher deductibles or copays for insurance or coverage disruptions for those living in persistent poverty areas, resulting in patient decisions to forgo surgery.^36^ Refusing surgery may also stem from personal beliefs, fear of surgery, or dissatisfaction with available treatment options.^37^ Future research could help shed light on the barriers and facilitators of surgery receipt for patients with CRC living in persistent poverty areas.

Our analysis also identified important variables that were not significant mediators. The types and quality of treatment were similar between those living in persistent and nonpersistent poverty areas, despite differences in the types of health insurance. Measures of disease severity or tumor aggressiveness were also similar between the residents of both types of areas, except for stage at diagnosis. While some variables were associated with risk of death (eg, lymphovascular invasion), these variables did not differ between those living in persistent poverty tracts and those living elsewhere and therefore could not be mediators.

### Strengths and Limitations

This study has several strengths. First, previous studies on persistent poverty have been descriptive and have not examined the reasons for the elevated mortality risk. Our identified reasons provide opportunities for interventions to reduce the risk among patients with CRC who live in these areas. Second, while several states have developed APCD, Arkansas is one of only a few states that have linked APCD to the state’s cancer registry, which provides critical tumor-related information for cancer research. We also acknowledge the limitations of our study. First, there might be residual confounding (eg, income). Not all patients with CRC who lived in persistent poverty areas had low income themselves. Second, examining mediators at a single time point before or after diagnosis could underestimate the proportion mediated.^38^ Third, this analysis focused on urban areas in Arkansas, which may limit generalizability to other states. However, since the 1990s, persistent poverty areas in Arkansas have had the highest CRC mortality in the US, making our findings especially relevant.^39^ Fourth, other unmeasured individual-level factors (eg, appropriate surveillance, frailty, travel time) or social determinants of health mediators may play a role.^15,40,41^ Fifth, we only focused on patients’ residence at the time of diagnosis. Data about where they lived before or after diagnosis could have influenced our findings.

## Conclusion

In this cohort study of CRC patients in urban persistent poverty areas, more advanced stage, decreased receipt of surgery, and type of health insurance were key mediators of their increased risk of mortality. Future research could improve our understanding of the role played by these mediators, and this awareness may help inform targeted interventions to reduce the elevated risk of mortality in this population.

## Notes

Supplement 1. **eTable 1.** Comparing characteristics of patients in the main AACR cohort versus the continuous coverage cohort (linked AACR-APCD cohort) by persistent poverty status **eTable 2.** Comparing characteristics of patients with and those without continuous coverage in the main AACR cohort (linked AACR-APCD cohort) **eTable 3.** Proportion of the association between persistent poverty and risk of death mediated by stage at diagnosis, having surgery, and health insurance, among the cohort of patients with continuous coverage. **eFigure.** Flow diagram of patient selection and inclusion/exclusion criteria **eAppendix 1.** Venous thromboembolism codes **eAppendix 2.** Anastomotic leak codes
eReferences

Supplement 2.
Data Sharing Statement
