# Supplementary material for: Poverty and Mortality Risk in Patients With Colorectal Cancer
Source: JAMA Netw Open. 2026 Apr 10;9(4):e266303. doi: 10.1001/jamanetworkopen.2026.6303 (PMC13069459; doi:10.1001/jamanetworkopen.2026.6303)
Supplement: Supplement 1. — eTable 1. Comparing characteristics of patients in the main AACR cohort versus the continuous coverage cohort (linked AACR-APCD cohort) by persistent poverty status eTable 2. Comparing characteristics of patients with and those without continuous coverage in the main AACR cohort (linked AACR-APCD cohort) eTable 3. Proportion of the association between persistent poverty and risk of death mediated by stage at diagnosis, having surgery, and health insurance, among the cohort of patients with continuous coverage. eFigure. Flow diagram of patient selection and inclusion/exclusion criteria eAppendix 1. Venous thromboembolism codes eAppendix 2. Anastomotic leak codes eReferences [file jamanetwopen-e266303-s001.pdf]

Supplemental Online Content

Schootman M, Li C, Peng C, et al. Poverty and mortality risk in patients with colorectal cancer. *JAMA Netw Open*. 2026;9(4):e266303. doi:10.1001/jamanetworkopen.2026.6303

eTable 1. Comparing characteristics of patients in the main AACR cohort versus the continuous coverage cohort (linked AACR-APCD cohort) by persistent poverty status

eTable 2. Comparing characteristics of patients with and those without continuous coverage in the main AACR cohort (linked AACR-APCD cohort)

eTable 3. Proportion of the association between persistent poverty and risk of death mediated by stage at diagnosis, having surgery, and health insurance, among the cohort of patients with continuous coverage.

eFigure. Flow diagram of patient selection and inclusion/exclusion criteria

eAppendix 1. Venous thromboembolism codes

eAppendix 2. Anastomotic leak codes

eReferences

This supplemental material has been provided by the authors to give readers additional information about their work.

© 2026 Schootman M et al. JAMA Network Open.

eTable 1. Comparing characteristics of patients in the main AACR cohort versus the continuous coverage cohort (linked AACR-APCD cohort) by persistent poverty status.*

Characteristic	**Main cohort**			**Continuous coverage cohort**		
	Persistent poverty (n=617) %	Nonpersistent poverty (n=4,411) %	Total (N=5,028)	Persistent poverty (n=270) %	Nonpersistent poverty (n=1,970) %	Total (N=2,240)
**Potential confounders**						
Sex (male)	50.7	51.6	51.5	47.4	48.3	48.2
	P=0.70			P=0.77		
Age (mean, sd)	64.8 (12.6)	64.6 (13.8)	64.6 (13.7)	68.4 (11.5)	68.6 (12.8)	68.6 (12.6)
	P=0.67			P=0.74		
CRC <age 50	10.9	13.5	13.2	4.4	7.7	7.3
	P=0.07			P=0.05		
Race						
African American	42.6	10.0	14.0	44.8	8.5	12.9
White	51.4	86.7	82.4	49.6	89.4	84.6
Other	6.0	3.3	3.6	5.6	2.1	2.5
	P<0.001			P<0.001		
Marital status (married)	33.9	49.0	47.2	31.5	47.2	45.3
	P<0.001			P<0.001		
Comorbidity						
None	25.1	27.9	27.6	25.2	33.0	32.1
One	19.8	19.6	19.7	24.4	24.3	24.3
Two or more	32.3	26.4	27.1	43.7	37.3	38.0
Unknown	22.9	26.1	25.7	6.7	5.4	5.6
	P=0.01			P=0.05		
**Potential mediators**						
Disease severity/aggressiveness						

© 2026 Schootman M et al. JAMA Network Open.

Stage								
Local	27.7		26.1	26.3	32.6		28.8	29.3
Regional	30.2		34.1	33.6	29.3		36.7	35.8
Distant	24.2		18.9	19.6	22.6		18.0	18.5
Unknown	18.0		20.9	20.5	15.6		16.5	16.4
		P=0.005				P=0.05		
Lymphovascular invasion								
Yes	21.1		24.1	23.7	20.4		24.3	23.9
No	42.5		43.6	43.5	46.3		44.6	44.8
Unknown	36.5		32.3	32.8	33.3		31.1	31.3
		P=0.07				P=0.34		
Tumor grade								
Well-differentiated	8.1		5.3	5.7	8.9		5.3	5.8
Moderately differentiated	43.8		45.2	45.1	43.3		43.9	43.8
Poorly differentiated	7.3		10.6	10.2	6.7		9.7	9.3
Undifferentiated	0.5		0.5	0.5	1.1		0.5	0.6
Unknown	40.4		38.4	38.6	40.0		40.6	40.5
		P=0.01				P=0.06		
Tumor location								
Colon	71.0		70.4	70.5	77.0		73.0	73.5
Rectal	29.0		29.3	29.6	23.0		27.0	26.5
		P=0.75				P=0.16		
Treatment type								
Type of surgery								
No surgery	20.1		16.0	16.5	16.3		15.0	15.2
Local excision	7.0		5.5	5.7	7.0		5.3	5.5
Partial, total colectomy, proctocolectomy	68.9		74.2	73.6	72.2		75.7	75.3
Palliative	0.5		0.7	0.7	0.4		0.7	0.7
Unknown	3.6		3.6	3.6	4.1		3.3	3.4
		P=0.04				P=0.60		

© 2026 Schootman M et al. JAMA Network Open.

Radiation								
Yes	13.0		13.2	13.1	9.3		11.2	10.9
No	79.1		79.9	79.8	81.5		82.3	82.2
Unknown	7.9		6.9	7.0	9.3		6.6	6.9
		P=0.65				P=0.19		
Chemotherapy								
Yes	32.1		33.9	33.7	38.9		40.0	39.8
No	67.9		66.1	66.3	61.1		60.1	60.2
		P=0.38				P=0.74		
<u>Quality of treatment</u>								
Lymph nodes examined #								
12 or more	62.3		68.9	68.1	57.1		69.8	68.3
<12 nodes	32.3		26.4	27.1	36.7		25.6	26.9
Unknown	5.5		4.8	4.8	6.2		4.7	4.8
		P=0.001				P=0.01		
<u>Healthcare access</u>								
Readmission within 30 days								
Not readmitted	34.0		37.4	37.0	77.8		83.7	83.0
Unplanned	3.6		3.2	3.2	3.7		3.1	3.1
Planned	1.8		1.0	1.1	3.0		1.0	3.1
Unknown	60.6		58.6	58.7	14.4		10.3	12.7
		P=0.15				P=0.01		
Health insurance								
Uninsured	3.7		2.8	2.9	1.1		0.6	0.7
Medicaid only	10.5		6.0	6.5	1.5		2.0	1.9
Medicare	42.6		44.1	43.9	58.6		56.6	56.8
Private	30.3		37.8	36.9	23.2		29.4	28.7
Medicare & Medicaid	7.1		4.3	4.7	10.0		7.2	7.5
Unknown	5.7		5.0	5.0	5.6		4.2	4.3
		P<0.001				P=0.16		
<u>Complications</u>								

© 2026 Schootman M et al. JAMA Network Open.

Venous thromboembolism	4.7		3.1	3.3	5.6		3.6	3.8
		P=0.04				P=0.12		
Anastomotic leakage	9.1		9.8	9.7	11.5		11.3	11.2
		P=0.02				P=0.01		

* p-values are for the comparison of potential confounders/mediators between CRC patients who lived in persistent poverty tracts and those who lived in nonpersistent tracts.

# Among those who had surgery.

Other race includes American Indian or Alaska Native, Asian, Native Hawaiian or Other Pacific Islander, and Unknown / Not stated.

© 2026 Schootman M et al. JAMA Network Open.

eTable 2. Comparing characteristics of patients with and those without continuous coverage in the main AACR cohort (linked AACR-APCD cohort).

Characteristic	Main cohort but without continuous coverage (n=2240) %	Continuous coverage cohort (n=2788) %	Total (N=5,028)	P-value
**Potential confounders**				
Sex (male)	54.1	48.2	51.5	<0.001
Age (mean, sd)	61.4 (13.6)	68.6 (12.6)	64.6 (13.6)	<0.001
CRC <age 50	17.9	7.3	13.2	<0.01
Race				<0.001
African American	14.9	12.9	14.0	
White	80.6	84.6	82.4	
Other	4.5	2.5	3.6	
Marital status (married)	48.7	45.3	47.2	0.02
Comorbidity				<0.001
None	23.9	32.1	27.6	
One	15.9	24.3	19.7	
Two or more	18.4	38.0	27.1	
Unknown	41.8	5.6	25.7	
**Potential mediators**				
Disease severity/aggressiveness				
Stage				<0.001
Local	23.8	29.3	26.3	

© 2026 Schootman M et al. JAMA Network Open.

Regional	31.9	35.8	33.6	
Distant	20.4	18.5	19.6	
Unknown	23.9	16.4	20.5	
Lymphovascular invasion				0.13
Yes	23.6	23.9	23.7	
No	42.5	44.8	43.5	
Unknown	33.9	31.3	32.8	
Tumor grade				0.08
Well-differentiated	5.6	5.8	5.7	
Moderately differentiated	46.0	43.8	45.1	
Poorly differentiated	10.9	9.3	10.2	
Undifferentiated	0.5	0.6	0.5	
Unknown	37.1	40.5	38.6	
Tumor location				<0.001
Colon	68.0	73.5	70.5	
Rectal	32.0	26.5	29.6	
Treatment type				
Type of surgery				0.16
No surgery	17.5	15.2	16.5	
Local excision	5.9	5.5	5.7	
Partial, total colectomy, proctocolectomy	72.2	75.3	73.6	
Palliative	0.7	0.7	0.7	
Unknown	3.7	3.4	3.6	
Radiation				<0.001
Yes	14.9	10.9	13.1	
No	77.9	82.2	79.8	

© 2026 Schootman M et al. JAMA Network Open.

Unknown	7.2	6.9	7.0	
Chemotherapy				<0.001
Yes	28.7	39.8	33.7	
No	71.3	60.2	66.3	
<u>Quality of treatment</u>				
Lymph nodes examined #				<0.001
12 or more	69.8	57.1	68.3	
<12 nodes	25.6	36.7	26.9	
Unknown	4.7	6.2	4.8	
<u>Healthcare access</u>				
Readmission within 30 days				<0.001
Not readmitted	0.0	1.9	0.9	
Unplanned	3.3	3.1	3.2	
Planned	1.0	1.2	1.1	
Unknown	95.7	94.8	57.9	
Health insurance				<0.001
Uninsured	4.7	0.7	2.9	
Medicaid only	10.3	1.9	6.5	
Medicare	33.5	56.8	43.9	
Private	43.5	28.7	36.9	
Medicare & Medicaid	2.4	7.5	4.7	
Unknown	5.6	4.3	5.0	
<u>Complications</u>				
Venous thromboembolism	2.9	3.8	3.3	0.06
Anastomotic leakage	8.5	11.3	9.7	<0.001

© 2026 Schootman M et al. JAMA Network Open.

* p-values are for the comparison of potential confounders/mediators between CRC patients who lived in persistent poverty tracts and those who lived in nonpersistent tracts.

# Among those who had surgery.

Other race includes American Indian or Alaska Native, Asian, Native Hawaiian or Other Pacific Islander, and Unknown / Not stated.

© 2026 Schootman M et al. JAMA Network Open.

eTable 3. Proportion of the association between persistent poverty and risk of death mediated by stage at diagnosis, having surgery, and health insurance, among the cohort of patients with continuous coverage.

Mediator	% mediated	P-value for the indirect effect	No.
Stage at Diagnosis	24.6	0.06	2165
Type of Surgery	6.7	0.56	2240
Health insurance	2.1	0.63	2240

The hazard of death was 22% higher for CRC patients who lived in persistent poverty tracts than for patients who lived in other tracts in unadjusted analysis 1.22 (95% CI: 1.02; 1.49).

© 2026 Schootman M et al. JAMA Network Open.

eFigure. Flow diagram of patient selection and inclusion/exclusion criteria.

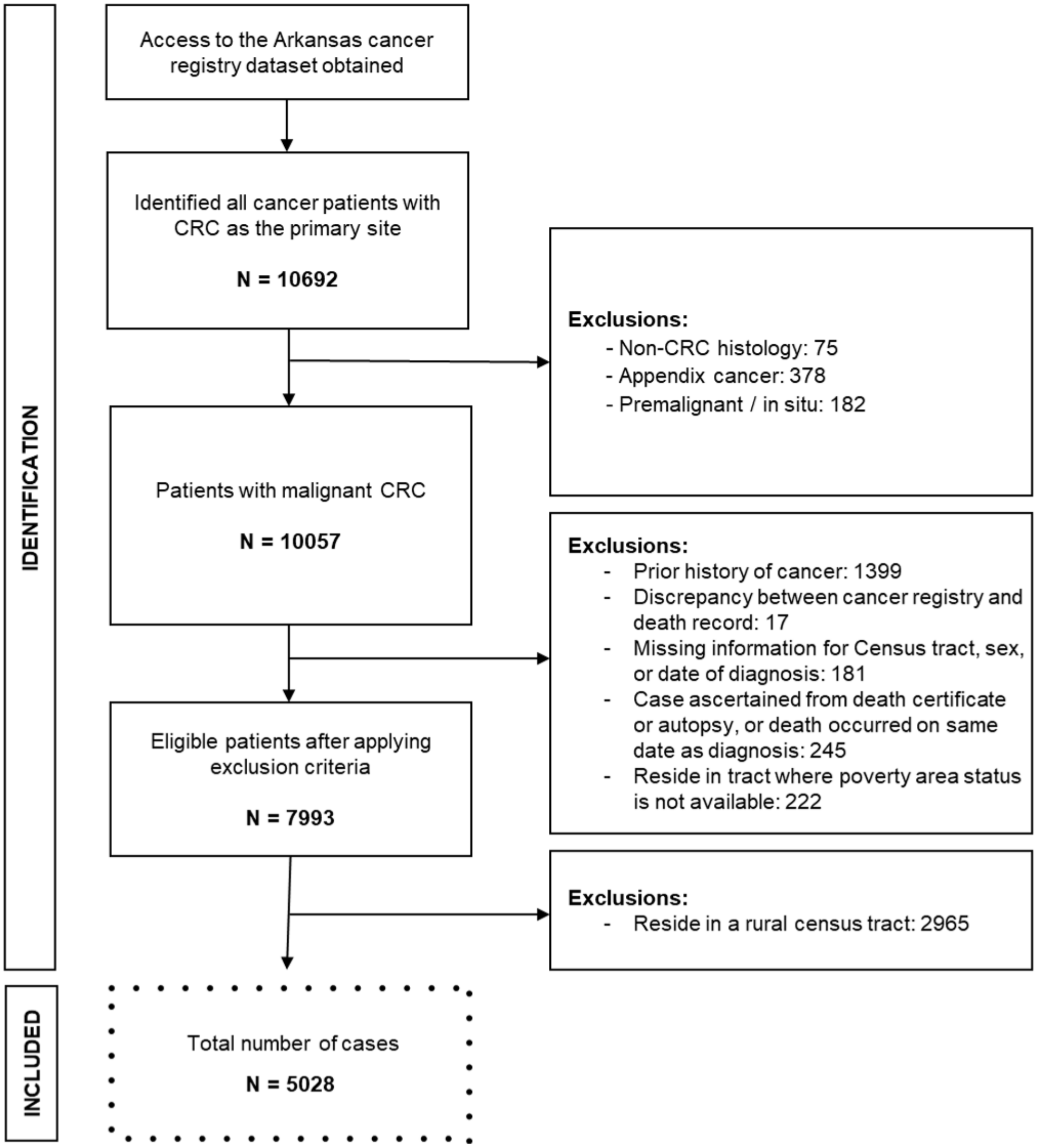

© 2026 Schootman M et al. JAMA Network Open.

eAppendix 1. Venous thromboembolism codes

Venous thromboembolism (VTE) is a healthcare challenge with significant morbidity, mortality, and healthcare costs.[1] The incidence of VTE following major abdominal or pelvic surgery ranges from 1 to 3% in real-world data, [2,3] with a significant proportion occurring after discharge.[4] VTE is also associated with significant mortality, following pulmonary embolism (PE) in cancer patients as high as 19%.[1,2]

We recently showed the variability of ICD code selection for VTE diagnosis in the published literature. We proposed a set of ICD-9-CM and ICD-10-CM codes for acute VTE definition after excluding isolated diagnoses of phlebitis, chronic DVT, and superficial venous thrombosis. These ICD codes showed greater accuracy than previous studies using inpatient and outpatient data.[5]

Appendix Table 1. List of applicable ICD-9-CM (2012 version) and ICD-10-CM (2015 version) codes.

ICD-9-CM code	ICD-10-CM code	Diagnosis
451	I80	Phlebitis & thrombophlebitis
451.0	I80.0x	Phlebitis and thrombophlebitis of superficial vessels of LEs
451.1	I80.2x	Phlebitis and thrombophlebitis of deep veins of LEs
451.11	I80.1x	Phlebitis and thrombophlebitis of femoral vein
451.19	I80.20x, I80.29x	Phlebitis and thrombophlebitis of deep veins of LEs, other
451.2	I80.3	Phlebitis and thrombophlebitis of LEs, unspecified
451.8	I80.8	Phlebitis and thrombophlebitis of other sites
451.81	I80.21x	Phlebitis and thrombophlebitis of iliac vein
451.82	I80.8	Phlebitis and thrombophlebitis of superficial veins of UEs
451.83	I80.8	Phlebitis and thrombophlebitis of deep veins of UEs
451.84	I80.8	Phlebitis and thrombophlebitis of UEs, unspecified
451.89	I80.8, I80.22x, I80.23x, I80.24x, I80.25x	Phlebitis and thrombophlebitis of other sites
451.9	I80.9	Phlebitis and thrombophlebitis of unspecified site
452	I81	Portal vein thrombosis
453	I82	Other venous embolism and thrombosis
453.0	I82.0	Budd-Chiari syndrome
453.1	I82.1	Thrombophlebitis migrans
453.2	I82.2x	Other VTE of inferior vena cava
453.3	I82.3	Other VTE of renal vein
453.4	I82.4	Acute VTE of deep vessels of LE
453.40	I82.40x, I82.49x	Acute VTE of unspecified deep vessels of LE
453.41	I82.41x, I82.42x, I82.4Yx	Acute VTE of deep vessels of proximal LE
453.42	I82.43x, I82.44x, I82.45x, I82.46x, I82.4Zx	Acute VTE of deep vessels of distal LE
453.5		Chronic VTE of deep vessels of LE
453.50	I82.5	Chronic VTE of unspecified deep vessels of LE
453.51	I82.50x, I82.59x	Chronic VTE of deep vessels of proximal LE
453.52	I82.51x, I82.52x, I82.5Yx	Chronic VTE of deep vessels of distal LE
453.6		VTE of superficial vessels of LE

© 2026 Schootman M et al. JAMA Network Open.

453.7<br>453.71<br>453.72<br>453.73<br>453.74<br>453.75<br>453.76<br>453.77<br>453.79<br>453.8<br>453.81<br>453.82<br>453.83<br>453.84<br>453.85<br>453.86<br>453.87<br>453.89<br>453.9	I82.53x, I82.54x, I82.55x, I82.56x,<br>I82.5Zx<br>I82.81x<br>I82.7<br>I82.71x<br>I82.72x<br>I82.70x<br>I82.A2x<br>I82.B2x<br>I82.C2x<br>I82.291<br>I82.891<br>I82.6<br>I82.61x<br>I82.62x<br>I82.60x<br>I82.A1x<br>I82.B1x<br>I82.C1x<br>I82.290<br>I82.890<br>I82.9x	Chronic VTE of other specified vessels<br>Chronic VTE of superficial veins of UE<br>Chronic VTE of deep veins of UE<br>Chronic VTE of UE, unspecified<br>Chronic VTE of axillary veins<br>Chronic VTE of subclavian veins<br>Chronic VTE of internal jugular veins<br>Chronic VTE of other thoracic veins<br>Chronic VTE of other specified veins<br>Acute VTE of other specified veins<br>Acute VTE of superficial veins of UE<br>Acute VTE of deep veins of UE<br>Acute VTE of UE, unspecified<br>Acute VTE of axillary veins<br>Acute VTE of subclavian veins<br>Acute VTE of internal jugular veins<br>Acute VTE of other thoracic veins<br>Acute VTE of other specified veins<br>Other VTE of unspecified site
415<br>415.0<br>415.1<br>415.11<br>415.12<br>415.13<br>415.19	I26<br>Not applicable<br>I26<br>Not applicable<br>126.01, I26.90<br>I26.02, I26.92<br>I26.09, I26.93, I26.94, I26.99	Acute pulmonary heart disease<br>Acute cor pulmonale<br>Pulmonary embolism and infarction<br>Iatrogenic pulmonary embolism and infarction<br>Septic pulmonary embolism<br>Saddle embolus of pulmonary artery<br>Other pulmonary embolism and infarction

The ICD-9-CM and ICD-10-CM code lists are publicly available at the Centers for Medicare & Medicaid Services website.
https://www.cms.gov/medicare/coding/icd9providerdiagnosticcodes/downloads/cmsv30_master_descriptions.zip
https://www.cms.gov/medicare/coding/icd10/downloads/2015-tables-index.zip
Abbreviations: VTE, venous embolism and thrombosis; LE, lower extremity; UE, upper extremity

© 2026 Schootman M et al. JAMA Network Open.

eAppendix 2. Anastomotic leak codes

Anastomotic leak (AL) is the most serious complication following colorectal surgery causing significant morbidity and mortality.[6] There are no specific medical codes to identify anastomotic leaks. The occurrence of anastomotic leaks was approximated through the presence of at least one of the ICD-9/ICD-10 diagnosis codes of infection, peritonitis, septicemia, or abscess.[7] These cases also needed to show the presence of at least one of the following procedures that are indicative of the anastomotic leak intervention: (1) laparotomy, (2) incision of abdominal wall, and (3) requirement of drainage during the index stay. We identified an AL during the hospital stay or up to 30 days post-discharge.[7]

**ICD-9: anastomotic leak case identification codes**	**ICD10**
041.04 Streptococcus infection in conditions classified elsewhere and of unspecified site, Group D [Enterococcus]	B95.2 Enterococcus as the cause of diseases classified elsewhere
041.49 Other and unspecified Escherichia coli [E. coli] unspecified site	B96.20 Unspecified Escherichia coli [E. coli] as the cause of diseases classified elsewhere<br><br>B96.29 Other Escherichia coli [E. coli] as the cause of diseases classified elsewhere
041.85 Other specified bacterial infections in conditions classified elsewhere and of unspecified site, other gram-negative organisms unspecified site	B96.89 Other specified bacterial agents as the cause of diseases classified elsewhere
539.81 Infection due to other bariatric procedure	K95.81 Infection due to other bariatric procedure
567.0 Peritonitis and retroperitoneal infections: peritonitis in infectious diseases classified elsewhere	K67 Disorders of peritoneum in infectious diseases classified elsewhere
567.21 Other suppurative peritonitis: peritonitis (acute) generalized	K65.0 Generalized (acute) peritonitis
567.22 Other suppurative peritonitis: peritoneal abscess	K65.1 Peritoneal abscess
569.5 Abscess of intestine	K63.0 Abscess of intestine
569.81 Fistula of intestine, excluding rectum and anus	K63.2 Fistula of intestine
569.83 Perforation of intestine	K63.1 Perforation of intestine (nontraumatic)
790.7 Bacteremia	R78.81 Bacteremia
998.31 Disruption of internal operation (surgical) wound	T81.32XA Disruption of internal operation (surgical) wound, not elsewhere classified, initial encounter
998.59 Other postoperative infection	K68.11 Postprocedural retroperitoneal abscess
998.6 Persistent postoperative fistula	T81.83XA Persistent postprocedural fistula, initial encounter
038.4 Septicemia due to other gram-negative organisms	x
038.8 Other specified septicemia	A41.89 Other specified sepsis
038.9 Unspecified septicemia	A41.9 Sepsis, unspecified organism
**At least one of the following procedure codes**	
54.11 Exploratory laparotomy (procedure code)	0DJ00ZZ Inspection of Upper Intestinal Tract, Open Approach<br>or:<br>0DJ60ZZ Inspection of Stomach, Open Approach<br>or:<br>0DJD0ZZ Inspection of Lower Intestinal Tract, Open Approach<br>or:

© 2026 Schootman M et al. JAMA Network Open.

	0DJU0ZZ Inspection of Omentum, Open Approach<br>or:<br>0DJW0ZZ Inspection of Peritoneum, Open Approach<br>or:<br>0WJG0ZZ Inspection of Peritoneal Cavity, Open Approach<br>or:<br>0WJJ0ZZ Inspection of Pelvic Cavity, Open Approach<br>or:<br>0WJP0ZZ Inspection of Gastrointestinal Tract, Open Approach<br>or:<br>0WJR0ZZ Inspection of Genitourinary Tract, Open Approach
54.12 Reopening of recent laparotomy site (procedure code)	0W3G0ZZ Control Bleeding in Peritoneal Cavity, Open Approach<br>or:<br>0W3H0ZZ Control Bleeding in Retroperitoneum, Open Approach<br>or:<br>0W3P0ZZ Control Bleeding in Gastrointestinal Tract, Open Approach<br>or:<br>0WJG0ZZ Inspection of Peritoneal Cavity, Open Approach<br>or:<br>0WJH0ZZ Inspection of Retroperitoneum, Open Approach<br>or:<br>0WJJ0ZZ Inspection of Pelvic Cavity, Open Approach
54.0 Incision of abdominal wall (procedure code)	0W9F00Z Drainage of Abdominal Wall with Drainage Device, Open Approach<br>or:<br>0W9F0ZZ Drainage of Abdominal Wall, Open Approach<br>or:<br>0W9H00Z Drainage of Retroperitoneum with Drainage Device, Open Approach<br>or:<br>0W9H0ZZ Drainage of Retroperitoneum, Open Approach<br>or:<br>0W9H40Z Drainage of Retroperitoneum with Drainage Device, Percutaneous Endoscopic Approach<br>or:<br>0W9H4ZZ Drainage of Retroperitoneum, Percutaneous Endoscopic Approach<br>or:<br>0WCJ3ZZ Extirpation of Matter from Pelvic Cavity, Percutaneous Approach<br>or:<br>0WCJ4ZZ Extirpation of Matter from Pelvic Cavity, Percutaneous Endoscopic Approach<br>or:<br>0WCP3ZZ Extirpation of Matter from Gastrointestinal Tract, Percutaneous Approach<br>or:<br>0WCP4ZZ Extirpation of Matter from Gastrointestinal Tract, Percutaneous Endoscopic Approach<br>or:<br>0WCR3ZZ Extirpation of Matter from Genitourinary Tract, Percutaneous Approach<br>or:<br>0WCR4ZZ Extirpation of Matter from Genitourinary Tract, Percutaneous Endoscopic Approach<br>or:

© 2026 Schootman M et al. JAMA Network Open.

	0WJF0ZZ Inspection of Abdominal Wall, Open Approach<br>or:<br>0WJH0ZZ Inspection of Retroperitoneum, Open Approach<br>or:<br>0Y9500Z Drainage of Right Inguinal Region with Drainage Device, Open Approach<br>or:<br>0Y950ZZ Drainage of Right Inguinal Region, Open Approach<br>or:<br>0Y9540Z Drainage of Right Inguinal Region with Drainage Device, Percutaneous Endoscopic Approach<br>or:<br>0Y954ZZ Drainage of Right Inguinal Region, Percutaneous Endoscopic Approach<br>or:<br>0Y9600Z Drainage of Left Inguinal Region with Drainage Device, Open Approach<br>or:<br>0Y960ZZ Drainage of Left Inguinal Region, Open Approach<br>or:<br>0Y9640Z Drainage of Left Inguinal Region with Drainage Device, Percutaneous Endoscopic Approach<br>or:<br>0Y964ZZ Drainage of Left Inguinal Region, Percutaneous Endoscopic Approach
54.19 Other laparotomy (procedure code)	0D9U00Z Drainage of Omentum with Drainage Device, Open Approach<br>or:<br>0D9U0ZZ Drainage of Omentum, Open Approach<br>or:<br>0D9V00Z Drainage of Mesentery with Drainage Device, Open Approach<br>or:<br>0D9V0ZZ Drainage of Mesentery, Open Approach<br>or:<br>0D9W00Z Drainage of Peritoneum with Drainage Device, Open Approach<br>or:<br>0D9W0ZZ Drainage of Peritoneum, Open Approach<br>or:<br>0W9G00Z Drainage of Peritoneal Cavity with Drainage Device, Open Approach<br>or:<br>0W9G0ZZ Drainage of Peritoneal Cavity, Open Approach<br>or:<br>0WCJ0ZZ Extirpation of Matter from Pelvic Cavity, Open Approach<br>or:<br>0WCP0ZZ Extirpation of Matter from Gastrointestinal Tract, Open Approach<br>or:<br>0WCR0ZZ Extirpation of Matter from Genitourinary Tract, Open Approach
54.91 Percutaneous abdominal drainage (procedure code)	0D9530Z Drainage of Esophagus with Drainage Device, Percutaneous Approach<br>or:<br>0D953ZZ Drainage of Esophagus, Percutaneous Approach<br>or:<br>0D9630Z Drainage of Stomach with Drainage Device, Percutaneous Approach<br>or:<br>0D963ZZ Drainage of Stomach, Percutaneous Approach

© 2026 Schootman M et al. JAMA Network Open.

	or:<br>0D9830Z Drainage of Small Intestine with Drainage Device, Percutaneous Approach<br>or:<br>0D983ZZ Drainage of Small Intestine, Percutaneous Approach<br>or:<br>0D9930Z Drainage of Duodenum with Drainage Device, Percutaneous Approach<br>or:<br>0D993ZZ Drainage of Duodenum, Percutaneous Approach<br>or:<br>0D9A30Z Drainage of Jejunum with Drainage Device, Percutaneous Approach<br>or:<br>0D9A3ZZ Drainage of Jejunum, Percutaneous Approach<br>or:<br>0D9B30Z Drainage of Ileum with Drainage Device, Percutaneous Approach<br>or:<br>0D9B3ZZ Drainage of Ileum, Percutaneous Approach<br>or:<br>0D9E30Z Drainage of Large Intestine with Drainage Device, Percutaneous Approach<br>or:<br>0D9E3ZZ Drainage of Large Intestine, Percutaneous Approach<br>or:<br>0D9H30Z Drainage of Cecum with Drainage Device, Percutaneous Approach<br>or:<br>0D9H3ZZ Drainage of Cecum, Percutaneous Approach<br>or:<br>0D9J30Z Drainage of Appendix with Drainage Device, Percutaneous Approach<br>or:<br>0D9J3ZZ Drainage of Appendix, Percutaneous Approach<br>or:<br>0D9N30Z Drainage of Sigmoid Colon with Drainage Device, Percutaneous Approach<br>or:<br>0D9N3ZZ Drainage of Sigmoid Colon, Percutaneous Approach<br>or:<br>0D9P30Z Drainage of Rectum with Drainage Device, Percutaneous Approach<br>or:<br>0D9P3ZZ Drainage of Rectum, Percutaneous Approach<br>or:<br>0D9Q30Z Drainage of Anus with Drainage Device, Percutaneous Approach<br>or:<br>0D9Q3ZZ Drainage of Anus, Percutaneous Approach<br>or:<br>0D9U30Z Drainage of Omentum with Drainage Device, Percutaneous Approach<br>or:<br>0D9U3ZZ Drainage of Omentum, Percutaneous Approach<br>or:<br>0D9U40Z Drainage of Omentum with Drainage Device, Percutaneous Endoscopic Approach<br>or:<br>0D9U4ZZ Drainage of Omentum, Percutaneous Endoscopic Approach

© 2026 Schootman M et al. JAMA Network Open.

	or:<br>0D9V30Z Drainage of Mesentery with Drainage Device, Percutaneous Approach<br>or:<br>0D9V3ZZ Drainage of Mesentery, Percutaneous Approach<br>or:<br>0D9V40Z Drainage of Mesentery with Drainage Device, Percutaneous Endoscopic Approach<br>or:<br>0D9V4ZZ Drainage of Mesentery, Percutaneous Endoscopic Approach<br>or:<br>0D9W30Z Drainage of Peritoneum with Drainage Device, Percutaneous Approach<br>or:<br>0D9W3ZZ Drainage of Peritoneum, Percutaneous Approach<br>or:<br>0D9W40Z Drainage of Peritoneum with Drainage Device, Percutaneous Endoscopic Approach<br>or:<br>0D9W4ZZ Drainage of Peritoneum, Percutaneous Endoscopic Approach<br>or:<br>0F9430Z Drainage of Gallbladder with Drainage Device, Percutaneous Approach<br>or:<br>0F9530Z Drainage of Right Hepatic Duct with Drainage Device, Percutaneous Approach<br>or:<br>0F953ZZ Drainage of Right Hepatic Duct, Percutaneous Approach<br>or:<br>0F9630Z Drainage of Left Hepatic Duct with Drainage Device, Percutaneous Approach<br>or:<br>0F963ZZ Drainage of Left Hepatic Duct, Percutaneous Approach<br>or:<br>0F9730Z Drainage of Common Hepatic Duct with Drainage Device, Percutaneous Approach<br>or:<br>0F973ZZ Drainage of Common Hepatic Duct, Percutaneous Approach<br>or:<br>0F9830Z Drainage of Cystic Duct with Drainage Device, Percutaneous Approach<br>or:<br>0F983ZZ Drainage of Cystic Duct, Percutaneous Approach<br>or:<br>0F9930Z Drainage of Common Bile Duct with Drainage Device, Percutaneous Approach<br>or:<br>0F9D30Z Drainage of Pancreatic Duct with Drainage Device, Percutaneous Approach<br>or:<br>0F9D3ZZ Drainage of Pancreatic Duct, Percutaneous Approach<br>or:<br>0F9G30Z Drainage of Pancreas with Drainage Device, Percutaneous Approach

© 2026 Schootman M et al. JAMA Network Open.

	or:<br>0F9G3ZZ Drainage of Pancreas, Percutaneous Approach<br>or:<br>0W9F30Z Drainage of Abdominal Wall with Drainage Device, Percutaneous Approach<br>or:<br>0W9F3ZZ Drainage of Abdominal Wall, Percutaneous Approach<br>or:<br>0W9F40Z Drainage of Abdominal Wall with Drainage Device, Percutaneous Endoscopic Approach<br>or:<br>0W9F4ZZ Drainage of Abdominal Wall, Percutaneous Endoscopic Approach<br>or:<br>0W9G30Z Drainage of Peritoneal Cavity with Drainage Device, Percutaneous Approach<br>or:<br>0W9G3ZZ Drainage of Peritoneal Cavity, Percutaneous Approach<br>or:<br>0W9G40Z Drainage of Peritoneal Cavity with Drainage Device, Percutaneous Endoscopic Approach<br>or:<br>0W9G4ZZ Drainage of Peritoneal Cavity, Percutaneous Endoscopic Approach<br>or:<br>0W9H30Z Drainage of Retroperitoneum with Drainage Device, Percutaneous Approach<br>or:<br>0W9H3ZZ Drainage of Retroperitoneum, Percutaneous Approach<br>or:<br>0W9J30Z Drainage of Pelvic Cavity with Drainage Device, Percutaneous Approach<br>or:<br>0W9J3ZZ Drainage of Pelvic Cavity, Percutaneous Approach

© 2026 Schootman M et al. JAMA Network Open.

eReferences

1. Lyman GH, Culakova E, Poniewierski MS, Kuderer NM. Morbidity, mortality and costs associated with venous thromboembolism in hospitalized patients with cancer. *Thromb Res*. 2018;164(1879-2472 (Electronic)):S112-S118.
2. Ross SW, Kuhlenschmidt KM, Kubasiak JC, et al. Association of the Risk of a Venous Thromboembolic Event in Emergency vs Elective General Surgery. *JAMA Surgery*. 2020;155(2168-6262 (Electronic)):503-511.
3. Gangireddy C, Rectenwald Jr Fau - Upchurch GR, Upchurch Gr Fau - Wakefield TW, et al. Risk factors and clinical impact of postoperative symptomatic venous thromboembolism. *J Vasc Surg*. 2007;45(0741-5214 (Print)):335-341.
4. Salous AA-O, Reyad A, Sweeney K, Mavanur A. A significant proportion of venous thromboembolism events in general surgical patients occurs after discharge: analysis of the ACS-NSQIP Essentials database. *Perioper Med*. 2019;8(2047-0525 (Print)):18.
5. Vyas V, Fuccello A, Corbin SL, Martin BC, Schootman M, Mavros MN. Venous thromboembolism diagnosis definition in claims data: implications for research. *Journal of Thrombosis and Thrombolysis*. 2025/12/01 2025;58(8):1141-1148. doi:10.1007/s11239-025-03125-y
6. Manwaring ML, Ko Cy Fau - Fleshman JW, Jr., Fleshman Jw Jr Fau - Beck DE, et al. Identification of consensus-based quality end points for colorectal surgery. *Diseases of the colon and rectum*. 2012;55(1530-0358 (Electronic)):294-301.
7. Lee SW, Gregory D, Cool CL. Clinical and economic burden of colorectal and bariatric anastomotic leaks. *Surg Endosc*. 2020;34(1432-2218 (Electronic)):4374-4381.

© 2026 Schootman M et al. JAMA Network Open.
