# Supplementary material for: Poverty and Mortality Risk in Patients With Colorectal Cancer
Source: JAMA Netw Open. 2026 Apr 10;9(4):e266303. doi: 10.1001/jamanetworkopen.2026.6303 (PMC13069459; doi:10.1001/jamanetworkopen.2026.6303)
Supplement: Supplement 2. — Data Sharing Statement [file jamanetwopen-e266303-s002.pdf]

Data Sharing Statement

Schootman. Poverty and Mortality Risk in Patients With Colorectal Cancer. *JAMA Netw Open*. Published April 10, 2026. doi:10.1001/jamanetworkopen.2026.6303

Data
Data available: No

Additional Information
Explanation for why data not available: The data are not allowed to be shared by the State of Arkansas
